# A novel sample holder for 4D live cell imaging to study cellular dynamics in complex 3D tissue cultures

**DOI:** 10.1038/s41598-018-28206-2

**Published:** 2018-06-29

**Authors:** D. Septiadi, J. Bourquin, E. Durantie, A. Petri-Fink, B. Rothen-Rutishauser

**Affiliations:** 1 Adolphe Merkle Institute, University of Fribourg, Chemin des Verdiers 4, 1700 Fribourg, Switzerland; 2 Department of Chemistry, University of Fribourg, Chemin du Musée 9, 1700 Fribourg, Switzerland

## Abstract

Three dimensional (3D) co-cultures to mimic cellular dynamics have brought significant impacts in tissue engineering approaches for biomedical research. Herein, we present a novel sample holder combined with time-lapse fluorescence imaging technique, referred as 4D live cell imaging, allowing direct visualization of various cells up to 24 hours. We further extended our approach to monitor kinetics and dynamics of particle uptake by cells and translocation across tissue membranes.

## Introduction

**Figure 1:**
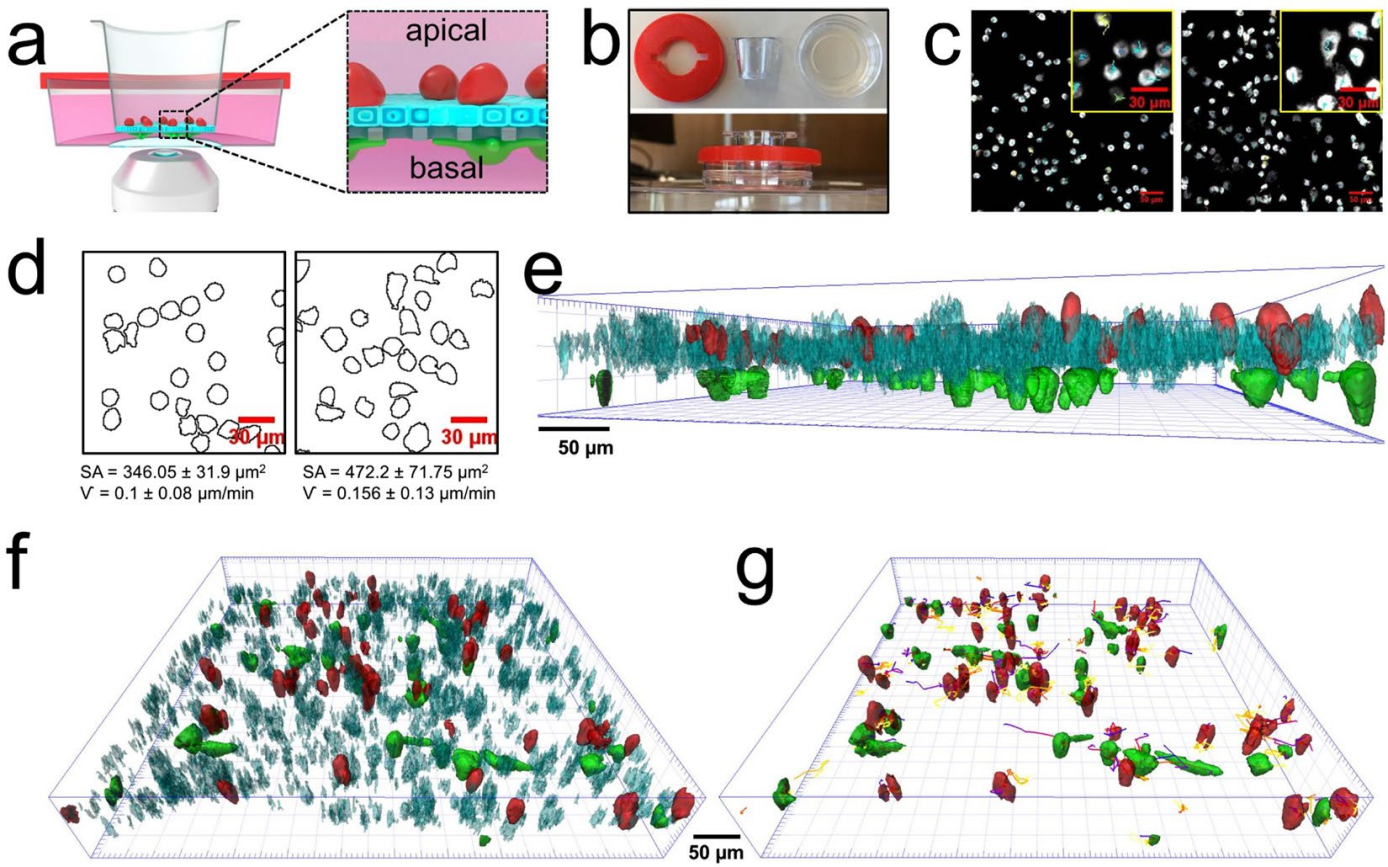
(**a**) A schematic representation of *in vitro* 3D lung co-culture model consisting of MDM (red), epithelial cells (cyan) and MDDC (green) cultured on permeable (porous) membrane insert with 3 µm pores (grey). Image was designed by Dr. Miguel Spuch-Calvar. (**b**) Photograph of designed insert holder (red), permeable inserts and glass bottom dish and their assembly. (**c**) Confocal micrographs of fluorescently-stained MDM cultured on apical (left) and basal (right). Scale bar 50 µm. Inset windows show enlarged area of the images in panel c and their respective migration tracks. (**d**) Outline of the cell mask of MDM cultured on apical (left) and basal (right) and their measured average cell surface area (SA) and velocity (ѵ) obtained by single cell analysis. (**e**) Side and (**f**) Top view of 3D reconstruction of 3D lung model. (**g**) Migration tracks of MDM and MDDC in lung co-culture model.

*In vitro* 3D co-culture models have offered many great advantages in the field of tissue engineering especially in providing more physiological environments and predictive output towards conventional 2D cultures^1,2^. Indeed it has been shown that cells cultured in a 3D configuration differ morphologically and physiologically^3^, and they possess more similar cellular behaviors to *in vivo* systems in comparison to their 2D culture counterparts^2^, allowing more realistic and reliable studies in a setting that resembles the *in vivo* environment^4^. A few examples have demonstrated the potential of 3D cell models in drug discovery^1^ or biopharmacokinetics study of nanodevices in specific organs^5–7^, where, these models have comprised of a combination of different cell types in a 3D scaffold comprised of biological polymers, and/or the co-culture of various cell types. In particular, 3D models of the human lung epithelial tissue barrier have been established which has allowed for the accurate *in vitro* simulation of bacterial airway infection^8^ and particulate uptake/translocation as well as cellular responses mimicking inhalation pathways^5^. Difficulties lay in the spatial characterization of thick *in vitro* tissues composed of several cell layers (i.e. >60 μm). Characterization of cell culture is normally performed by fluorescence light confocal microscopy, however, with 3D cell culture, the analytical challenge lies in the characterization of live co-culture/tissue in real time. Solving this will allow for the deeper understanding of the behavior of cells grown in a 3D co-culture configuration, as well as the study of the kinetics and dynamics of fluorescently-labelled drugs or nanocarriers over a longer period of time. In this brief communication, we report the design and fabrication of a sample holder by 3D printing technology optimized for 3D cell culture models cultured on widely used and commercially available permeable membrane inserts, combined with a simple time-lapse fluorescence confocal imaging technique, which we refer to as 4D live cell imaging. The system allows for the direct visualization of a live 3D cell model, without the need for the removal of membrane inserts and cell fixation. By using this method, the uptake and translocation of fluorescently-labelled silica particles across cellular tissue barriers has been followed in a 3D co-culture lung model consisting of three cell types namely, epithelial cells and macrophages on the apical and dendritic cells on the basolateral side of permeable inserts^9–11^ over several hours up to one day (Fig. 1a).

The design of the imaging chamber consists of an insert holder fabricated by 3D printing with a “twist-fastener” lock mechanism (see methods for details) and a commercially available and widely used glass bottom dish (Fig. 1b, Supplementary Fig. 1). The system was engineered to hold or hang the permeable inserts and maintain a minimal distance between the bottom of the insert and the glass bottom dish in order to keep the cells on the lower side of the insert membrane alive. The distance of less than 0.5 mm allows for the use of typical long working distance objective lenses on the microscope. In the first instance, in order to investigate the applicability of the designed system, 2D monocultures of monocyte-derived macrophages (MDM) were cultured on either the apical or basolateral side of permeable inserts and labelled with a fluorescence dye for live cell tracking. Imaging acquisitions were performed using a confocal laser scanning microscope with 20X magnification lens (working distance 0.55 mm and numerical aperture 0.8). The image was acquired in a z-stack and in time-lapse mode (with the slice thickness of  2 μm and a time frame of 15 min). Figure 1c displays the final output represented as mean fluorescence intensity images revealing the dynamics (e.g. cell movement) of live MDM both in apical and basolateral configuration (see Supplementary Video 1). Single cell analysis shows the cells cultured on basal side (i.e. hanging cells) possessed a different morphology (i.e. more elongated shape and higher surface area, SA) than in the apical side (Fig. 1d) but no significant difference in terms of motility speed (ѵ) for cells cultured in both orientation was observed (ѵ *ca*. 0.1 µm/min).

This technique was then applied to characterize systems that are more complex: 3D co-cultures. A 3D lung model was constructed according to a previously reported procedure^10–12^, where the system resembles human lung epithelial tissue. This barrier consists of three different types of cells, namely, human epithelial type-II cells (A549), MDM (on the apical side of the insert), and monocytes-derived dendritic cells (MDDC; on the basal side; Fig. 1a) and the cell type specific response within this model has been determined previously via multicolor flow cytometry^11^. Each cell type was fluorescently labeled with different fluorophores to distinguish between the cells and the corresponding emission channels were recorded sequentially to avoid any signal overlap (see Methods section). The fastest scanning rate for the three channels and 35–40 slices (slice thickness 2 μm) was *ca*. 3–4 min. To avoid any cell stress and possible light-induced cell killing due to extended light exposure, the time frame was increased to 20 min and the imaging was performed for up to one day. The obtained raw data was processed and rendered using a 3D rendering software for the better visualization of cells.

Our study shows, to the best of our knowledge, the behavior of living cells in a 3D co-culture model resembling the human lung epithelial tissue barrier for the first time (Supplementary Video 2). The three different cell types are easily distinguished (Fig. 1e,f) by the different fluorophores. The intensity of the fluorophores for live cell tracking was not reduced during the experiments and it was always possible to clearly identify the cells. Moreover, both MDM (red) and MDDC (green) are primary non-proliferating cells while epithelial cells in differentiated tissue have a slow proliferation rate, hence the intensity of the fluorophores were not reduced due to proliferation. The movement of MDM and MDDC was followed over 24 h (see corresponding tracks in Fig. 1g and Supplementary Video 2). In particular for MDM, the measured motility speed is 0.18 ± 0.14 µm/min, i.e. *ca*. two times faster than MDM’s movement (i.e. 0.1 ± 0.08 µm/min) cultured only on the insert. We hypothesized that this difference is twofold. Firstly, the phagocytotic nature of macrophages to clear debris from dead cells in the surrounding: the MDM are compelled to move and clean the cell debris from the apoptotic epithelial cells. The difference in velocity can be also attributed to substrate stiffness, i.e. insert *vs*. epithelial cell carpet. It is important to note that only a few cells underwent apoptosis, however, no significant reduction of the cell number was observed during the imaging experiment indicating the effect of long acquisition time can be well tolerated with higher time frame. In addition, we have performed cytotoxicity test based on lactate dehydrogenase (LDH) assay and we found out that cells were still viable even after the imaging experiment (Supplementary Fig. 2).

**Figure 2:**
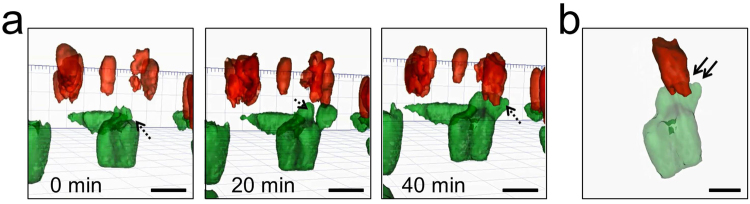
4D live cell imaging reveals MDDC-MDM contact in the 3D lung model. (**a**) Time lapse images showing formation of MDDC’s protrusion (green, dashed arrows) upon contact with MDM (red). (**b**) MDDC-MDM communication through cellular contact (solid arrows). Scale bar 20 µm.

MDM and MDDC are the most prolific immune cells in the respiratory tract, where their movements within the lung epithelia are essential to their function. Macrophages are professional phagocytotic cells, whereas dendritic cells are antigen-presenting cells which can take up antigens both within and directly below the surface epithelium by extending protrusions into the respiratory lumen^13^. Upon activation, the dendritic cells migrate to the draining lymph nodes and interact with T-cells^14,15^. Hence, it was our aim to be able to visualize parts of this process (e.g. uptake of antigens and vertical transmigration across the epithelial layer to the dendritic cells) in real time. During the image acquisition, no vertical movement of macrophages from apical to basolateral side nor of dendritic cells from basal to apical side was observed (Supplementary Video 2). However, we were able to capture and reconstruct the establishment of cellular contact between MDDC and MDM within the epithelial layer (Fig. 2a) which so far has been only shown in 3D *in vitro* using fixed tissue imaging^10^. Our time-lapse data provide a mechanistic explanation of the establishment of contact between the immune cell types which was initiated by the formation of membrane protrusion by MDDC, followed by mechanical interaction between the two cells and retraction of the contact (Fig. 2b). The duration of the contact between two cells occurred *ca*. 40 min (see Supplementary Video 3).

**Figure 3:**
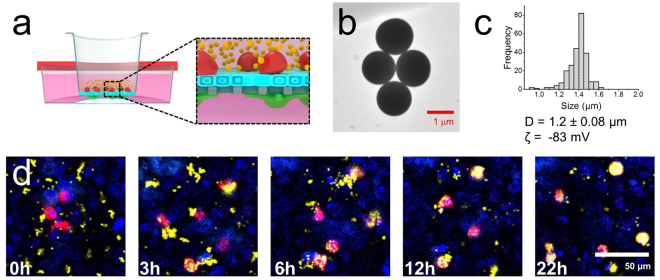
(**a**) Schematic representation of particle uptake experiment of in 3D lung model. Image was designed by Dr. Miguel Spuch-Calvar. (**b**) Transmission electron micrograph of 1.2 µm rhodamine B-labeled silica particles. (**c**) Graph showing size distribution of silica particles analyzed by TEM. D and ζ denotes diameter and zeta potential of particles, respectively. (**d**) Time-lapse micrographs showing cellular uptake of silica particles (yellow) by MDM (red) on A549 epithelial carpet (blue).

**Figure 4:**
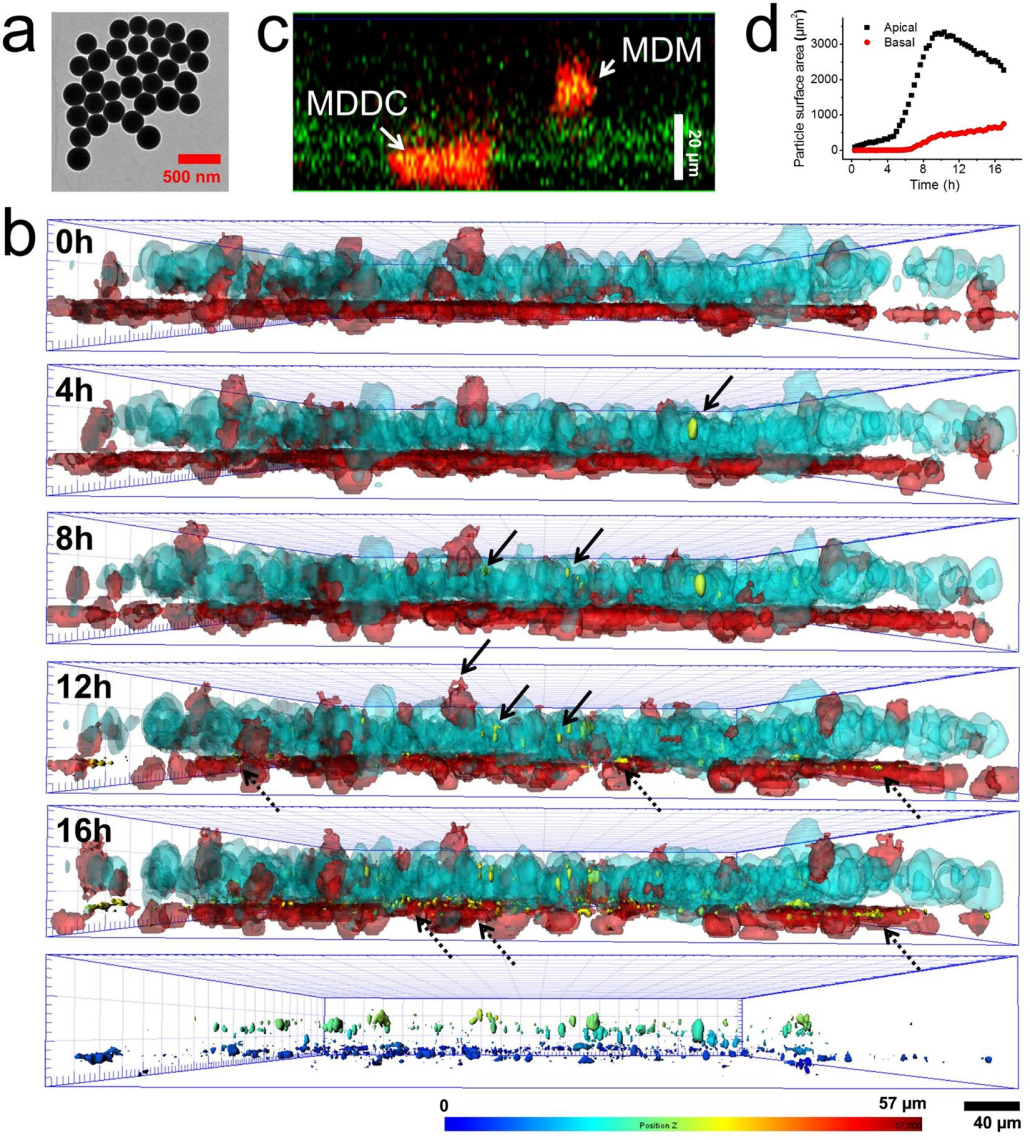
(**a**) Transmission electron micrograph of 260 nm rhodamine B-labeled silica particles. (**b**) Kinetics of cellular uptake and translocation of silica particles (yellow) from apical to basal side visualized by 4D live cell imaging. Red color denotes MDM (apical) or MDDC (basal) while cyan represents epithelial A549 cells. Solid arrows represent internalized particles by MDM or A549 cells and dashed arrows show translocated particles in basal side. Bottom panel shows particles 16 h post incubation which are color-coded based on their spatial positions. (**c**) Orthogonal view of confocal micrographs showing internalization of particles (green) by MDM (red) in apical side as well as by MDDC (red) in basal side. (**d**) Semi quantitative image analysis showing particle number in apical and basal side over time. The presence of particles in basal side was hypothesized due to the uptake of translocated particles by MDDC.

Our approach was further extended to visualize cellular uptake and translocation of particles within the 3D lung model (Fig. 3a). Previous fixed imaging data of co-culture models have shown the internalization and translocation of particles both for singular or aggregated forms from apical side (macrophages and epithelial cells) to basolateral one (dendritic cells)^9,12,16^, however the biokinetics of these processes have never been visualized in real time. To validate first the ability of our system in monitoring particle uptake, we exposed our lung model to large size rhodamine B-labeled silica particles (1.2 µm in diameter, see material characterization in Fig. 3b,c) to ease visualization of the particles. To avoid any signal overlap with rhodamine B, the MDM and MDDC were labelled using the same fluorophore (i.e. Vybrant^®^ DiD) and their distinction was only detected by their position (basal *vs*. apical). Figure 3d shows the kinetic of cellular uptake of silica particles (yellow) by MDM (red) on A549 epithelial carpet (blue). As can be seen, majority of the particles were internalized by the MDM after 22 h, and very few particles by epithelial cells. We also noticed that only small numbers of particles were translocated to MDDC site which can be associated to reduction of particle translocation due to their large size (Supplementary Fig. 3a**)**. This result is in agreement with an earlier finding where polystyrene particles (1 µm in size) were found more in MDM rather than in epithelial cells or MDDC in the same co-culture model^12^. The 3D lung model was further exposed to smaller size rhodamine B-labeled silica particles (260 nm in diameter, Fig. 4a) at the initial concentration of 20 µg/mL (see material characterization for the particles in Supplementary Fig. 3b, Electronic Supporting Information). The kinetics of cellular uptake of silica particles (yellow) by MDM and epithelial cells (Supplementary Video 4) was recorded. Both cell types in the apical sides (i.e. epithelial and macrophages) internalized the particles as can be seen from z-stack experiments (Fig. 4b,c and Supplementary Fig. 3c). For the first time, the translocation kinetics of silica particles from apical side (i.e. MDM and epithelial cells) was visualized, passing through the membrane insert, to basal side (MDDC; Fig. 4b). Semi quantitative image analysis of the amount of particles in both the apical and basal side (which is expressed as particle surface area) show first a slow (0–5 h), and then significant (5–10 h) increase of particle intensity at the apical side indicating the uptake of particles by MDM and epithelial cells. Meanwhile, the earliest particle presence in basal side was detected only *ca*. 7–8 h post particle incubation indicating translocation and this signal, as expected, was found increasing over time (Fig. 4d). This provides a reasonable explanation of a route of particle translocation as the presence of free particles was often found in basal side before they were uptaken/phagocytosed by MDDC (Supplementary Fig. 4). We hypothesized that these free particles might be originally from externalized products by epithelial cells, i.e. exocytosed particles. This finding presents another particle uptake pathway by MDDC, building upon the possible extension of cytoplasmic processes or through particle transfer from MDM to MDDC as reported previously for polystyrene particles^10^.

In summary, a novel sample holder has been designed and built by 3D printing, and combined with 3D cell imaging allowing for the direct visualization of a live co-culture model that mimics human lung epithelial tissues. The developed system is robust, does not need any cellular fixation or insert membrane removal, and hence can provide a simple and useful platform to study many cellular processes of 3D cell models including dynamics of cell adhesion, transmigration, wound healing, immune response, etc. i.e. in the presence of nanoparticles or bacteria mimicking infection.

## Methods

### Design of insert holder

The design of the live imaging chamber consists of two main components. First is a donut-shaped cap with a “twist-fastener” lock mechanism (outer diameter 40 mm, inner diameter 16.6 mm, length vs width of opener 4.3 mm × 3 mm) which is designed specifically for polyethylene terephthalate (PET) transparent BD Falcon permeable inserts (growth area of 0.9 cm^2^, PET membranes for 12-well plates, pore size 3.0 µm in diameter; BD Biosciences). To design the cap we used the open-source program FreeCAD, which was subsequently 3D printed on a UltiMaker 2+ (UltiMaker, The Netherlands). The material we used was polylactic acid polymer. Before the imaging experiment the holder was autoclaved for sterilization purpose. Second component is a commercially available and widely used glass bottom dish (Mattek Inc, US).

### Preparation of co-culture model

The 3D co-culture model consisted out of three different types of cells namely human alveolar epithelial type II cell line (A549) which was obtained from the American Type Culture Collection (ATCC, USA), human blood monocyte-derived macrophages (MDM) and dendritic cells (MDDC) which were isolated from buffy coats provided by the blood donation service SRK Bern and purified using CD14 Microbeads (Milteny Biotech) following the procedure reported previously^17^. The cells were grown in cell culture media containing RPMI 1640 (Gibco, Life Technologies Europe B.V., Zug, Switzerland) supplemented with 10% (v/v) fetal bovine serum (FBS; PAA Laboratories, Chemie Brunschwig AG, Basel, Switzerland), 1% (v/v) L-Glutamine (Life Technologies Europe) and 1% (v/v) penicillin/streptomycin (Gibco) and kept in a humidified incubator (37 °C, 5% CO_2_) until reaching 90% cell confluency of T-75 culture flask (Thermo Fisher Scientific, Germany). The co-culture models were prepared as previously described^10^. Shortly, A549 cells (5.10^5^ cells/mL, 0.5 mL, apical side) were seeded on a PET transparent BD Falcon permeable inserts (growth area of 0.9 cm^2^, pore size 3.0 µm in diameter, PET membranes for 12-well plates; BD Biosciences) placed in a 12 well plates BD Falcon tissue culture plates (BD Biosciences) containing 1.5 mL medium (lower chamber). Cells were cultured for 4 days and the medium was changed after the 2nd day. On day 5, medium was removed from the apical and basolateral chambers and the monolayer was stained with nuclei stained Hoechst 33342 (Invitrogen) or Vybrant^®^ DiO (Thermo Fisher Scientific, Germany) following the protocols provided by manufacturers for 30 min. The layer was washed three times with PBS. The inserts were gently turned up-side down, placed in a petri dish and possible cells grown on the basolateral side of the membrane were gently removed with a cell scraper. MDDC (8.10^5^ cells/mL, 65 µL) were priorly stained with Vybrant^®^ DiI (Thermo Fisher Scientific, Germany) for 15 min and washed in PBS, were then pipetted onto the basolateral side of the inserts and incubated for 70 min. The inserts containing A549 and MDDC were held in the 3D printed insert holder and the bottom part was placed in the glass bottom dish containing 1.5 mL medium. MDM (4.10^4^ cells/mL, 0.5 mL) which were pre-labeled with Vybrant^®^ DiD (Thermo Fisher Scientific, Germany) for 15 min and washed in PBS, were added on the apical side (*i.e*. the top of A549) and the MDM were let to sediment for 30 min before the imaging experiment.

### Synthesis and characterization of rhodamine B-labeled silica particles

Two different sizes of silica particles were synthesized following the Stöber method previously described in literature^18^. For small size particles, shortly, 9 mL of the silica precursor (i.e. tetraethyl orthosilicate, TEOS; Sigma Aldrich, Germany) was added to a preheated (60 °C) mixture of 100 mL of ethanol, 18 mL of deionized water and 14 mL of ammonium hydroxide (Sigma Aldrich, Germany). After 1 min of core formation, 300 µL of (3-aminopropyl) triethoxysilane (APTES; Sigma Aldrich, Germany) – rhodamine B conjugate, prepared the previous day by mixing 7.5 µL of APTES with 528 µL of rhodamine B isothiocyanate in ethanol (10 mg/mL) and stirred overnight, was added to the mixture to form fluorescently-label layers around these initially formed cores. The reaction was further stirred overnight and purified by centrifugation at 5,000 g and washed with ethanol three times followed by redispersion in autoclaved milliQ water 3 times. For larger particles, 2 mL of TEOS was added dropwise (2 mL/h) at room temperature to a mixture of 75 mL of isopropanol, 25 mL of methanol and 21 mL of ammonium hydroxide. After 1 hour of core formation, premixed solution of TEOS (6 mL) and APTES rhodamine B isothiocyanate (300 µL) was added dropwise (2 mL/h) to the reaction mix. The reaction was further stirred overnight and purified by centrifugation at 100 g and washed with ethanol two times followed by redispersion in autoclaved milliQ water 3 times. The synthesized particles were then visualized using a transmission electron microscope (FEI Tecnai Spirit, US) and their corresponding size was determined using FIJI software (NIH, US). The hydrodynamic diameter and zeta potential were measured by dynamic light scattering and zeta potential analyzer (Brookhaven, US), respectively. The particle concentration was determined by measuring the weight of 2 mL of particle suspension after evaporating the water at 50 °C.

### Cellular uptake experiment

The experiment was performed by incubating the 3D lung model with 260 nm or 1.2 µm rhodamine B-labeled silica particles at the initial concentration 20 µg/mL or 50 µg/mL in 500 µL of cell culture media. After particle addition, the imaging experiment was immediately conducted.

### Fluorescence imaging

All of the fluorescence images were acquired using Zeiss LSM 710 confocal laser scanning inverted microscope set up with 20X magnification, numerical aperture, NA, 0.8 of Zeiss LCI Plan-NEOFLUAR objective lens (Zeiss GmbH, Germany). Different fluorophores (Hoechst 33342, Vybrant^®^ DiI, rhodamine B, and Vybrant^®^ DiD) were excited sequentially at 405, 541 and 633 nm and their emissions were collected correspondingly by the detector with the frame size 512 pixel × 512 pixel. The image was acquired in a z-stack and in time-lapse mode with the slice thickness 2 μm and time between each frames 15–20 min. Image processing (*i.e*. mean intensity projection) was carried out directly using Zen 2010 software (Zeiss GmbH). 3D rendering was performed using Imaris (Bitplane, Switzerland). False color images were adjusted to better distinguish different types of cells and nanoparticles.

### Image analysis

Single cell (surface) analysis was performed using self-written macro and 2D cell tracking was analysed by TrackMate plugin in Fiji software (NIH, US). 3D cell tracking was performed in Imaris. Semi quantitative image analysis of particle uptake (particle surface area measurement) in apical and basal side was performed using Fiji and Matlab (MathWorks, US). Shortly, the fluorescence channel of particles was classified depending on their position (basal *vs*. apical). Using sum slice projection in Fiji the corresponding time-lapse z-stack images of particles was reconstructed. Particle surface area (in µm^2^) was calculated through measurement of pixel area in the entire single frame after intensity thresholding and binarization and it was plotted against the incubation time (different time frame).

### Data availability

Experimental data are available from the corresponding author upon reasonable request.

## Electronic supplementary material

Supplementary Information

Supplementary Video 1

Supplementary Video 2

Supplementary Video 3

Supplementary Video 4

## References

[1] Edmondson R, Broglie JJ, Adcock AF, Yang L (2014). Three-dimensional cell culture systems and their applications in drug discovery and cell-based biosensors. Assay Drug Dev. Technol..

[2] Lee J, Cuddihy MJ, Kotov NA (2008). Three-dimensional cell culture matrices: state of the art. Tissue Eng. Part B Rev..

[3] Baharvand H, Hashemi SM, Kazemi Ashtiani S, Farrokhi A (2006). Differentiation of human embryonic stem cells into hepatocytes in 2D and 3D culture systems *in vitro*. Int. J. Dev. Biol..

[4] Justice BA, Badr NA, Felder RA (2009). 3D cell culture opens new dimensions in cell-based assays. Drug Discov. Today.

[5] Rothen-Rutishauser BM, Kiama SG, Gehr P (2005). A three-dimensional cellular model of the human respiratory tract to study the interaction with particles. Am. J. Respir. Cell Mol. Biol..

[6] Ricketts KPM (2014). A 3D *In vitro* cancer model as a platform for nanoparticle uptake and imaging investigations. Small.

[7] Goodman TT, Ng CP, Pun SH (2008). 3-D tissue culture systems for the evaluation and optimization of nanoparticle-based drug carriers. Bioconjugate Chem..

[8] Ding P, Wu H, Fang L, Wu M, Liu R (2014). Transmigration and phagocytosis of macrophages in an airway infection model using four-dimensional techniques. Am. J. Respir. Cell Mol. Biol..

[9] Durantie E (2017). Biodistribution of single and aggregated gold nanoparticles exposed to the human lung epithelial tissue barrier at the air-liquid interface. Part. Fibre Toxicol..

[10] Blank F, Rothen-Rutishauser B, Gehr P (2007). Dendritic cells and macrophages form a transepithelial network against foreign particulate antigens. Am. J. Respir. Cell Mol. Biol..

[11] Clift MJ (2017). A novel technique to determine the cell type specific response within an *in vitro* co-culture model via multi-colour flow cytometry. Sci. Rep..

[12] Blank F (2011). Macrophages and dendritic cells express tight junction proteins and exchange particles in an *in vitro* model of the human airway wall. Immunobiology.

[13] Rescigno M (2001). Dendritic cells express tight junction proteins and penetrate gut epithelial monolayers to sample bacteria. Nat. Immunol..

[14] Stoll S, Delon J, Brotz TM, Germain RN (2002). Dynamic imaging of T cell-dendritic cell interactions in lymph nodes. Science.

[15] Miller MJ, Wei SH, Parker I, Cahalan MD (2002). Two-photon imaging of lymphocyte motility and antigen response in intact lymph node. Science.

[16] Kuhn DA (2015). Cellular uptake and cell-to-cell transfer of polyelectrolyte microcapsules within a triple co-culture system representing parts of the respiratory tract. Sci. Technol. Adv. Mater..

[17] Steiner S (2013). Comparison of the toxicity of diesel exhaust produced by bio- and fossil diesel combustion in human lung cells *in vitro*. Atmospheric Environ..

[18] Stöber W, Fink A, Bohn E (1968). Controlled growth of monodisperse silica spheres in the micron size range. J. Colloid Interface Sci..

