# Supplementary material for: A novel sample holder for 4D live cell imaging to study cellular dynamics in complex 3D tissue cultures
Source: Sci Rep. 2018 Jun 29;8:9861. doi: 10.1038/s41598-018-28206-2 (PMC6026119; doi:10.1038/s41598-018-28206-2)
Supplement: Supplementary file 1 — Supplementary Information [file 41598_2018_28206_MOESM1_ESM.pdf]

Electronic Supporting Information

A novel sample holder for 4D live cell imaging to study cellular dynamics in complex 3D tissue cultures

D. Septiadi[1,*], J. Bourquin[1], E. Durantie[1], A. Petri-Fink[1,2], B. Rothen-Rutishauser[1,*]

[1]Adolphe Merkle Institute, University of Fribourg, Chemin des Verdiers 4, 1700 Fribourg, Switzerland

[2]Department of Chemistry, University of Fribourg, Chemin du Musée 9, 1700 Fribourg, Switzerland

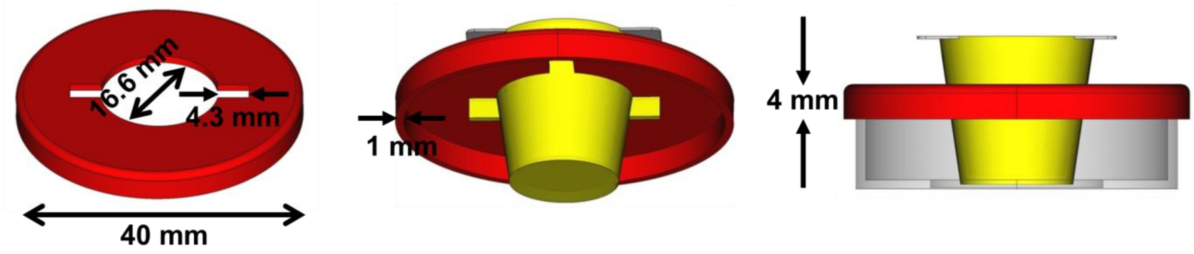

Supplementary Fig. 1. Design of a sample holder consisting of a permeable insert (yellow), insert holder (red) and a glass bottom dish (grey) and their assembly.

LDH-based cytotoxicity assay. In a total of 600 µL of cell media was collected from basal and apical side of the 3D co-culture model before the imaging experiment started. 600 µL of fresh cell culture media was added to the sample and the imaging experiment as described was conducted for 12h. Cells were placed inside incubator for another 12h before another 600 µL of cell media was collected. Negative control sample was prepared by culturing the cells only inside incubator for 24h. The release of lactate dehydrogenase (LDH) as an indicator of membrane impairment into the supernatant was evaluated using a commercially available LDH diagnostic kit (Roche Applied Science, Mannheim, Germany), following the manufacturer's protocol. Determination of the enzyme activity was performed photometrically by measuring the absorbance at 490 nm (reference wavelength at 630 nm). Cells exposed to 0.2% Triton X-100 for ca. 1 hour were used as a positive control. Cytotoxicity value was calculated following this expression:

$$Cytotoxicity\ (\%) = \frac{experimental\ \text{value of the endpoint} - \text{negative control}}{\text{positive control} - \text{negative control}} * 100$$

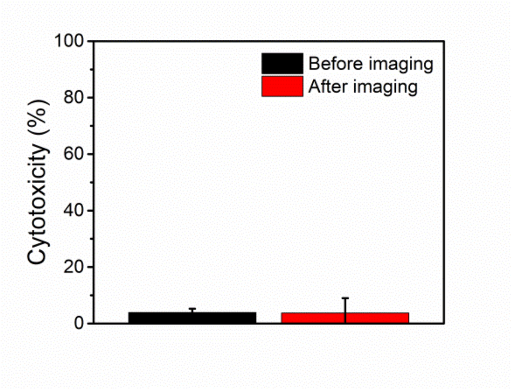

Supplementary Fig. 2. LDH cytotoxicity profile before and after imaging.

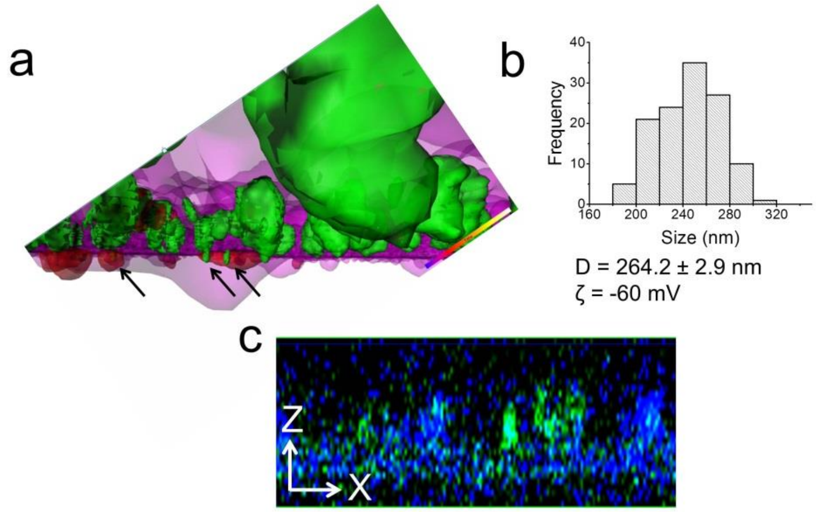

Supplementary Fig. 3.a. 3D render showing the presence of particles (green, black arrow) in MDDC (red; basal side). **b**. Graph showing size distribution of silica particles analyzed by TEM. Measured hydrodynamics diameter (D) and zeta potential (ζ) of the particles in water are 262.2 ± 2.9 nm and -60 mV, respectively. **c**. Orthogonal view of confocal micrographs showing internalization of particles (green) by epithelial cells (blue) in apical side.

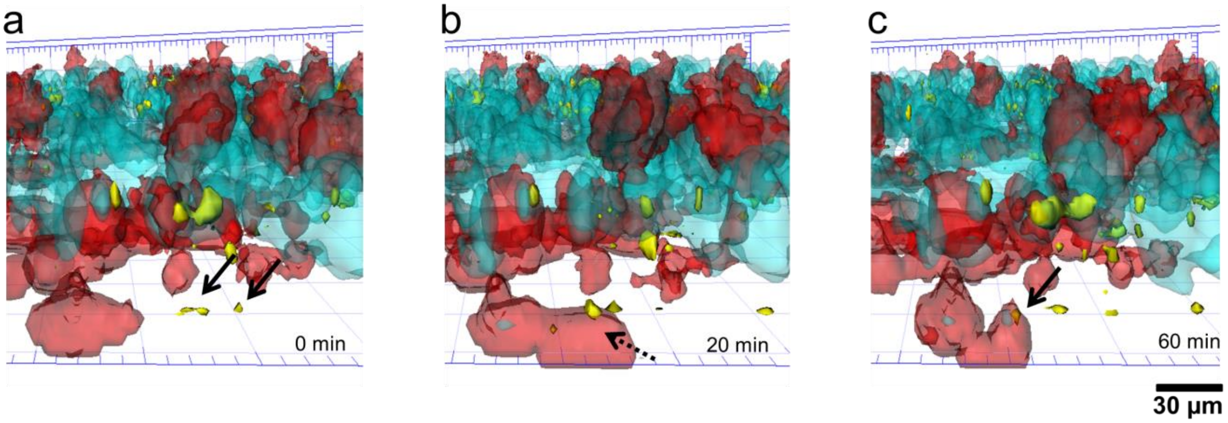

Supplementary Fig. 4. Time-lapse micrographs displaying particle uptake and translocation in 3D lung model after 11 h of incubation. a. Presence of free particles (solid arrow) in basal side before they were uptaken/phagocytosed by MDDC. b. Formation of protrusion (dashed arrow) by MDDC (red) during particle uptake. c. Internalized particles in MDDC (solid arrow) followed by cell retraction.

Videos

Supplementary Video 1. Dynamics of living MDM (grey color) cultured both in apical and basal configuration. Images were acquired for each 15 mins. 2D cell tracking was performed using TrackMate plugin in Fiji software.

Supplementary Video 2. 4D live cell imaging shows dynamics of the 3D lung model. The co-culture model consisting of MDM (red), epithelial cells (cyan) and MDDC (green) cultured on permeable (porous) membrane insert. Time-lapse and z-stack images were processed (including 3D rendering and cell tracking) using Imaris software. Acquisition time is 20 mins. (Video) frame rate is 29 frames/s.

Supplementary Video 3. MDDC-MDM contact in the 3D lung model. MDDC cells are shown in green and MDMs in red. Acquisition time is 20 mins. (Video) frame rate is 29 frames/s.

Supplementary Video 4. Kinetics and dynamics of particle uptake and translocation in the 3D lung model. Red color denotes MDM (apical) or MDDC (basal) while cyan represents epithelial A549 cells. Yellow color shows silica particles. Acquisition time is 20 mins. (Video) frame rate is 29 frames/s.
